# Neurodevelopmental Progression and Functional Outcomes in a Child With Joubert Syndrome: A Case Study

**DOI:** 10.7759/cureus.92677

**Published:** 2025-09-18

**Authors:** Tomás Ferrão, Rita Alvelos, Kátia Mauricio, Sónia Almeida, Sandra Rebimbas

**Affiliations:** 1 Department of Pediatrics, Unidade Local de Saúde da Região de Aveiro, Aveiro, PRT

**Keywords:** developmental and behavioural pediatrics, genetic analysis, joubert syndrome (js), methylphenidate (mph), motor skills, neuro-rehabilitation, pediatric brain mri

## Abstract

Joubert syndrome (JS) is a rare autosomal recessive neurodevelopmental disorder characterized by malformations of the cerebellum and brainstem, most notably the pathognomonic “molar tooth sign” on magnetic resonance imaging (MRI). Clinical manifestations are heterogeneous and include dysmorphic features, motor and ocular abnormalities, and, in most patients, intellectual developmental disorder (IDD). We describe the case of a nine-year-old boy with prenatal suspicion of ventricular asymmetry and postnatal findings of macrocephaly and facial dysmorphisms. In infancy, he exhibited nystagmus, oculomotor apraxia, irregular breathing, ataxia, and delayed motor milestones; MRI at 18 months revealed cerebellar vermis hypoplasia with a “molar tooth sign,” supporting the diagnosis of JS despite the absence of a causative variant in extended genetic testing. Over time, motor and coordination deficits improved with sustained physical and occupational therapy, complemented by school and family support. Cognitive abilities remained within the expected range, although expressive language delay and motor coordination difficulties were present. At the age of eight years, he was diagnosed with attention-deficit/hyperactivity disorder, inattentive subtype, and responded well to methylphenidate, with marked improvements in attention, concentration, handwriting, and academic performance. This case illustrates a milder neurological phenotype of JS with preserved learning abilities, emphasizing the importance of MRI in diagnosis and highlighting the benefit of early individualized rehabilitation and targeted treatment of comorbidities. It also underscores the role of genetic counseling, although no pathogenic variants were identified, reflecting the syndrome’s genetic diversity.

## Introduction

Joubert syndrome (JS) is a rare autosomal recessive neurodevelopmental disorder characterized by congenital malformations of the cerebellum and brainstem. The hallmark radiological feature is the "molar tooth” sign seen on magnetic resonance imaging (MRI), which results from hypoplasia of the cerebellar vermis, thickened and horizontally oriented superior cerebellar peduncles, and a deepened interpeduncular fossa [1,2].

The clinical spectrum of JS is heterogeneous. Common early manifestations include neonatal hypotonia, developmental delay, ataxia, abnormal ocular movements such as nystagmus or oculomotor apraxia, and irregular respiratory patterns including episodic apnea and tachypnea [2,3]. Cognitive outcomes vary widely: while almost all patients present with intellectual disability, rarely preserved cognition or only mild delays were also described [3]. Motor coordination deficits are nearly universal due to cerebellar involvement, and neuropsychiatric comorbidities have been increasingly recognized, including attention deficit hyperactivity disorder (ADHD) and autism spectrum disorder (ASD) [4].

Multisystem involvement may also occur, including retinal dystrophy, renal anomalies, hepatic fibrosis, and polydactyly, but some patients exhibit isolated neurological findings without systemic manifestations [1]. Early recognition based on neuroimaging allows prompt initiation of supportive interventions, such as physical, occupational, and speech therapies, as well as targeted pharmacological treatment for comorbid conditions. Understanding the variable clinical course and the potential for functional improvement is essential to guide long-term care and therapeutic management [1,4].

## Case presentation

The patient is a nine-year-old boy with JS who is followed at a Regional Center’s Neurodevelopment Outpatient Clinic. The diagnosis was established in early childhood. The child was born to a secundigravida, primiparous mother with a history of one previous miscarriage. Serologic screenings during pregnancy were unremarkable. Fetal ultrasound at 21 weeks of gestation revealed ventricular asymmetry, and fetal karyotyping was normal. He was delivered by cesarean section due to failure to progress in labor, with Apgar scores of 9, 10, and 10 at one, five, and ten minutes, respectively.

**Figure 1:**
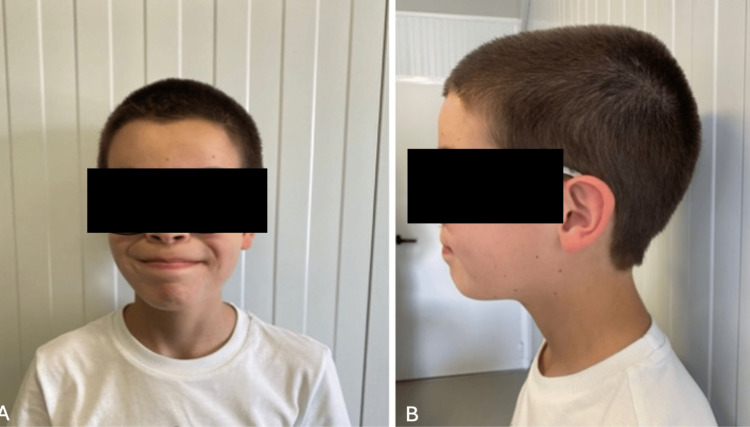
Patient's facial dysmorphism Patient’s front (A) and profile (B) pictures at age nine, in which anteverted nostrils and dolichocephaly are visible. Written informed consent was obtained from the patient’s legal guardian for publication of this case report and accompanying images

At birth, his weight was 3950 g (89th percentile), length 52 cm (78th percentile), and head circumference 37 cm (96th percentile). During follow-up, his head circumference continued to grow above the 97th percentile, while his weight and height progressed within normal percentiles. On physical examination, macrocephaly was noted along with distinct facial features, including anteverted nostrils and dolichocephaly, which were evident from infancy and remain noticeable at nine years of age (Figure 1).

During infancy, he developed multiple clinical signs that prompted an etiological investigation. An intermittent horizontal nystagmus was observed during the first weeks of life, in addition to lateral head movements. Although less frequent by seven months of age, ophthalmologic evaluation revealed lateral head movements during visual fixation, consistent with oculomotor apraxia. Motor milestone delays were also apparent: at 21 months, he was unable to walk independently, displayed signs of ataxia, and required support from objects to ambulate. At that age, other neurodevelopmental domains were appropriate for his age. Parents also reported intermittent respiratory irregularities with episodes suggestive of sleep apnea during the first year of life.

**Figure 2:**
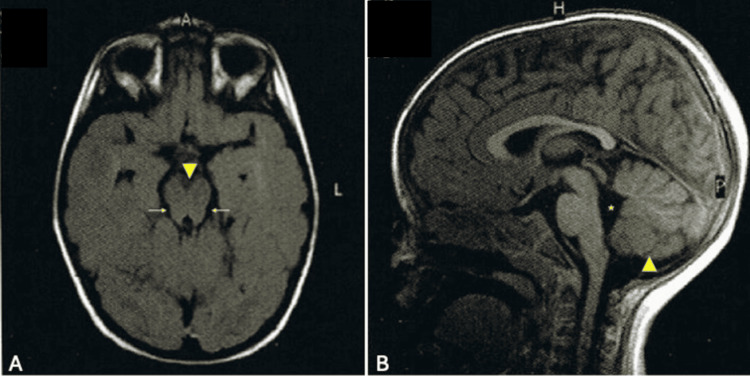
Brain MRI performed at 18 months of age (A) Axial T1-weighted image demonstrates a deepened interpeduncular fossa (arrowhead) and a “molar tooth sign” with slightly elongated, thickened, and horizontally oriented superior cerebellar peduncles (arrows), although not in its most typical form in Joubert syndrome. (B) Sagittal T1-weighted image evidences hypoplasia of the cerebellar vermis, with poor delineation of its lower portion (arrowhead), and enlargement of the fourth ventricle (asterisk).

A brain MRI performed at 18 months of age revealed hypoplasia of the cerebellar vermis and a mildly elevated fastigium. The fourth ventricle displayed a “molar tooth” sign, consistent with JS, besides cerebellum hypoplasia and fourth ventricle enlargement (Figure 2).

Following evaluation by the Genetics department, targeted sequencing of an expanded panel of genes associated with genetic disorders was performed, but no pathogenic variants were identified. Although molecular confirmation was not obtained, the diagnosis of JS was supported by the clinical and imaging findings. Alternative diagnoses such as Dandy-Walker malformation, rhombencephalosynapsis, pontocerebellar hypoplasia, and isolated congenital ocular motor apraxia were excluded based on imaging findings and normal neonatal metabolic screening, with the presence of the pathognomonic molar tooth sign confirming JS. Additionally, abdominal ultrasound showed no signs of renal or hepatic malformations, and periodic ophthalmologic evaluations revealed no evidence of retinal involvement.

The child remains under follow-up at his local Neurodevelopment Clinic. In addition to early motor delays, particularly in gait acquisition, the patient demonstrated difficulties with fine motor coordination. Fine motor skills were especially affected, prompting referral to physical and occupational therapy at around 24 months of age. These interventions were provided consistently and were later supplemented by school-based support measures.

Speech development was within expected milestones, though mild expressive language difficulties were noted early on, primarily due to impulsivity. He received ongoing speech therapy, which contributed to improved verbal articulation and communication skills. He has remained fully verbal, with adequate language comprehension and expression for his age.

At the age of six, the patient began formal schooling and quickly demonstrated learning potential. Nonetheless, by age eight, he presented with inattention symptoms that began to interfere with academic performance. A diagnosis of ADHD, inattentive subtype, was made. Pharmacological treatment with methylphenidate was initiated, starting at 5 mg daily (0.15 mg/kg/day) and later titrated to 10 mg twice daily (0.5 mg/kg/day). Following this intervention, his attention span and academic engagement improved significantly, especially in reading, mathematics, handwriting, and task completion.

A school report at age nine described his overall learning abilities as adequate, with very high performance in verbal memory (digit span) and reading fluency above the 90th percentile. Nonetheless, difficulties in abstract reasoning, text composition, and sustained attention were still evident. Improvements were noted in handwriting legibility and motor control following both therapeutic and pharmacologic intervention, with documented calligraphic differences before and after methylphenidate initiation.

Importantly, the patient's learning abilities have remained within the expected range, with no need for formal neuropsychological assessment. The family was consistently engaged in therapeutic routines at home, contributing to his steady progress. At present, he attends the fourth grade, with reasonably age-adjusted academic performance and good school integration. Current challenges include mild motor coordination issues and sustained attention deficits, for which he continues to receive psychological support and educational accommodations. The mother also reports a persistent, subtle head-shaking movement, particularly noticeable during tasks requiring concentration, such as reading, or while walking, especially when changing direction.

A summary of the patient’s clinical signs and their age of onset is presented in Table 1.

**Table 1:** Summary of clinical features and age of onset ADHD: attention deficit hyperactivity disorder

Clinical Feature	Age of Onset	Notes/Comments
Macrocephaly	Birth	Head circumference ≥96th percentile
Dysmorphic features	Infancy	Dolichocephaly; anteverted nares
Nystagmus (intermittent)	First weeks	Horizontal; resolved during the first year of life
Irregular breathing (apneas)	Infancy	Intermittent; mostly during sleep
Oculomotor apraxia	7 months	Mainly observed during visual fixation
Ataxia / gait delay	Until 24 months	Required support for ambulation up to 24 months
Fine motor coordination delay	Early childhood	Handwriting and manual dexterity difficulties; improved with methylphenidate
ADHD (inattentive subtype)	8 years	Treated with methylphenidate
Subtle head shaking	Persistent	More evident during concentration or when changing direction in gait

## Discussion

This case illustrates a milder neurological phenotype of JS, with no significant cognitive difficulties reported and adequate learning abilities for age and meaningful gains in development following early therapeutic interventions. This case also underscores that early recognition of JS often hinges on clinical manifestations beginning as early as the neonatal or infancy periods. Hypotonia, oculomotor apraxia, and delayed motor milestones are among the most distinctive and early-presenting signs of JS; however, their diagnostic significance can be overshadowed by later-emerging symptoms. Clinicians should prioritize these clinical features, along with characteristic neuroimaging findings, to facilitate earlier genetic testing and interventions, thereby potentially improving long-term outcomes. Importantly, there were no signs of systemic involvement, such as retinal, renal, or hepatic anomalies, which are known to negatively influence prognosis [5].

Although intellectual disability is reported in the majority of JS patients, a subset, particularly those without multisystem disease, may show only mild delays or even age-appropriate cognitive abilities [6]. In our patient, formal academic assessments at the age of nine years confirmed adequate school performance, with reading fluency, verbal memory, and basic arithmetic skills within or above expected levels. Mild difficulties in abstract reasoning and written expression were attributed largely to attentional deficits rather than cognitive delay.

Attention difficulties emerged during early schooling and led to a diagnosis of ADHD, predominantly inattentive type. This is in line with recent literature that highlights a growing recognition of neuropsychiatric comorbidities in JS, including ADHD and ASD [7,8]. In this case, treatment with methylphenidate resulted in improved concentration and academic output, including marked gains in handwriting quality, suggesting that stimulant therapy may be both safe and effective in selected JS patients.

Motor coordination deficits, which reflect underlying cerebellar defects, are nearly universal in JS [9]. Our patient showed delayed independent walking and persistent fine motor difficulties. Nonetheless, his gross motor skills improved significantly with early and sustained physical and occupational therapy. Handwriting, in particular, benefited from a combined approach of motor rehabilitation and pharmacological treatment for ADHD. Verbal development was initially affected by impulsivity and reduced motor planning but progressed to fluent, age-appropriate expression with regular speech therapy.

The long-term outlook in JS is closely tied to early developmental gains and the presence or absence of systemic complications [10]. In this case, the absence of organ involvement and the child’s access to structured rehabilitation services likely contributed to his positive trajectory. The consistent involvement of caregivers, particularly the mother’s involvement in therapeutic strategies at home, was also crucial and should be acknowledged as a modifiable factor in long-term outcome and the importance of caregivers’ therapeutic involvement.

A key limitation of this case is the absence of molecular confirmation despite genetic testing. Although the patient underwent targeted sequencing using an expanded gene panel, specifically a disease exome, no pathogenic variants were identified. This underscores a known challenge in JS, as current panels may not capture all causative genes, intronic variants, or structural rearrangements [9,11]. Additionally, some variants may remain classified as variants of uncertain significance (VUS) [1,2].

Over 35 genes have been associated with JS, and in approximately 10-30% of clinically diagnosed cases, no molecular diagnosis is reached, particularly in milder phenotypes [2,3]. Therefore, while the clinical and radiological findings, notably the “molar tooth” sign, strongly supported the diagnosis, the lack of genetic confirmation limits genotype-phenotype correlation, prognostic clarity, and precise genetic counseling for recurrence risk.

Furthermore, although the patient’s cognitive evolution was closely monitored and appeared largely age-appropriate, formal neuropsychological testing was not conducted. This may have limited the depth of objective assessment of attention deficits and executive function beyond classroom and clinical observations.

Lastly, while the positive response to multidisciplinary rehabilitation and pharmacologic treatment is notable, this report reflects a single case and should be interpreted with caution regarding broader generalizability. Longitudinal follow-up is still ongoing, and while current outcomes are encouraging, they may not necessarily predict functioning in adolescence or adulthood.

## Conclusions

This case underscores the heterogeneity of JS and reinforces the value of early diagnosis, individualized rehabilitation, and targeted interventions for associated conditions such as ADHD. It also highlights the importance of long-term neurodevelopmental follow-up, not only to monitor emerging symptoms but also to adapt support strategies as the child grows.

Ultimately, this report adds to the limited but growing literature on JS cases with favorable cognitive outcomes, emphasizing that, despite its rarity and complexity, a positive functional prognosis is attainable in selected patients.
